# HIT-6 and EQ-5D-5L in patients with migraine: assessment of common latent constructs and development of a mapping algorithm

**DOI:** 10.1007/s10198-021-01342-9

**Published:** 2021-07-10

**Authors:** Ana Sofia Oliveira Gonçalves, Dimitra Panteli, Lars Neeb, Tobias Kurth, Annette Aigner

**Affiliations:** 1 Institute of Public Health, Charité - Universitätsmedizin, Berlin, Charitépl. 1, 10117 Berlin, Germany; 2 Department of Health Care Management, Technische Universität Berlin, Berlin, Germany; 3 Department of Neurology, Charité - Universitätsmedizin Berlin, Berlin, Germany; 4 Institute of Biometry and Clinical Epidemiology, Charité - Universitätsmedizin Berlin, Berlin, Germany; 5 Berlin Institute of Health (BIH), Anna-Louisa-Karsch-Straße 2, 10178 Berlin, Germany

**Keywords:** Mapping, EQ-5D, QALY, Utilities, HIT-6, Migraine, I1, C3

## Abstract

**Objective:**

The aims of this study were to assess whether there is a conceptual overlap between the questionnaires HIT-6 and EQ-5D and to develop a mapping algorithm allowing the conversion of HIT-6 to EQ-5D utility scores for Germany.

**Methods:**

This study used data from an ongoing randomised controlled trial for patients suffering from migraine. We assessed the conceptual overlap between the two instruments with correlation matrices and exploratory factor analysis. Linear regression, tobit, mixture, and two-part models were used for mapping, accounting for repeated measurements, tenfold cross-validation was conducted to validate the models.

**Results:**

We included 1010 observations from 410 patients. The EQ-5D showed a substantial ceiling effect (47.3% had the highest score) but no floor effect, while the HIT-6 showed a very small ceiling effect (0.5%). The correlation between the instruments’ total scores was moderate (− 0.30), and low to moderate among each domain (0.021–0.227). The exploratory factor analysis showed insufficient conceptual overlap between the instruments, as they load on different factors. Thus, there is reason to believe that the instruments’ domains do not capture the same latent constructs. To facilitate future mapping, we provide coefficients and a variance–covariance matrix for the preferred model, a two-part model with the total HIT-6 score as the explanatory variable.

**Conclusion:**

This study showed that the German EQ-5D and the HIT-6 lack the conceptual overlap needed for appropriate mapping. Thus, the estimated mapping algorithms should only be used as a last resort for estimating utilities to be employed in economic evaluations.

**Supplementary Information:**

The online version contains supplementary material available at 10.1007/s10198-021-01342-9.

## Introduction

Migraine is a common neurological condition affecting 10.6% of the German population (one-year prevalence) [1]. It is associated with comorbidities such as psychiatric disorders (depression and anxiety, among others), respiratory disorders, and chronic pain, and it leads to a significant reduction in quality of life [2, 3].

This condition also imposes an economic burden on health care systems due to increased demand for goods and services and work-related productivity losses [4, 5]. As healthcare systems face the challenge of limited resources, economic evaluations provide tools for decision-makers to analyse competing alternatives—in terms of both costs and consequences [6]. Cost-utility analyses, a form of economic evaluation, measure consequences with generic measures of health gain, commonly expressed in quality-adjusted life years (QALYs) [6]. The EuroQol five-dimensional questionnaire (EQ-5D) is a generic utility-based instrument which allows the estimation of utility scores, and thus, the calculation of country-specific QALYs [7]. It analyses five different dimensions: mobility, self-care, usual activities, pain/discomfort, and anxiety/depression. The initial version of EQ-5D had only three levels within each dimension, while the improved EQ-5D-5L, henceforth EQ-5D-5L will be referred to as EQ-5D, has five levels, while maintaining the same five dimensions. The levels indicate no problems (1), slight problems (2), moderate problems (3), severe problems (4), and unable to/extreme problems (5). Health states are defined by combining digits for the five dimensions, enabling 3125 possible health states. Health states can be represented with five-digit codes or converted using country-specific single index values.

Clinical trials in migraine often use monthly migraine days as a primary endpoint and the International Headache Society actually recommends the use of monthly migraine days as the primary endpoint for HTAs involving preventive treatments [8]. Where generic preference-based measures are not deemed ideal, other approaches include using condition-specific instruments or condition-specific preference-based measures (to our knowledge there is none for migraine). However, analyses with disease-specific instruments do not allow decision-makers to compare resource allocation across different conditions.

Nevertheless, several trials in the migraine field (e.g. [9, 10]) only collect migraine-specific health-related quality-of-life (HRQOL) instruments, but do not collect generic preference-based ones, which can be used to conduct cost-utility analyses. There are several migraine-specific HRQOL questionnaires such as the Headache Impact Test-6 (HIT-6), the Migraine Disability Assessment (MIDAS), and the Migraine-Specific Quality-of-Life Questionnaire (MSQ). The HIT-6 is a headache-specific questionnaire, which evaluates how headaches affect someone’s ability to function on the job, at school, at home, and in social situations [11]. This instrument does not have a preference-based scoring system, thus it does not permit the calculation of QALYs. Mapping overcomes this issue by providing an algorithm which allows the estimation of QALYs even if a preference-based HRQOL instrument was not included in the study. However, to perform mapping between two instruments, there should be a conceptual overlap between them. ISPOR guidelines on mapping state that these algorithms can only be successful if there is sufficient overlap between the analysed instruments [12]. Although the selected instruments do not have to measure the same symptoms or functional (dis)abilities, they do need to address the same underlying concepts.

One study by Gillard et al. has already mapped the EQ-5D to the HIT-6, but used quality weights for England in a Brazilian population [13]. Several studies have shown the impact of using different country-specific value sets of EQ-5D on the interpretation of results [14, 15]. Furthermore, the authors used variables in their algorithm which are not always collected in trials, such as ethnicity. A large number of trials which do not involve drugs but e.g. behavioural interventions often do not collect ethnic information (e.g. [16–19]). Applying the validation method of splitting the available data set into two has been criticised because of its limited ability to depict the uncertainty in the results and increased bias in the performance estimates in proportionally large test sets [20].

Based on these considerations, we will address the issue of whether there is enough conceptual overlap between the two instruments using not only correlation tables, but also exploratory factor analysis (EFA). Based on regression-type approaches we will develop a mapping function to predict EQ-5D utility values for Germany from HIT-6 values, including variables widely used in migraine trials, and validate them with tenfold cross-validation.

## Materials and methods

### Data

This study is based on data from the SMARTGEM project. SMARTGEM is an ongoing national randomised controlled clinical trial, which seeks to assess if a digital intervention via the use of a headache app and online consultations leads to a decrease in migraine frequency. The intervention consists of a certified medical app where patients document trigger factors, attacks, and medication in an electronic calendar; the app analyses the diary and evaluates trigger factors and proposes individually tailored treatment plans; a web-based tool where patients communicate both with other patients and specialists. HIT-6 and EQ-5D were completed by all users at baseline and after 3, 6, 9, and 12 months. Registration ID in the German clinical trials register is DRKS00016328.

### Statistical analyses

#### Conceptual overlap

To analyse the strength of the relationship between HIT-6 scores and EQ-5D domains, correlation coefficients accounting for repeated measurements were computed. We also examined the capacity of each instrument to detect changes in HRQOL over time, referred to as responsiveness, by computing standardised response mean(s) (SRM). SRM is defined as a ratio of the difference in the mean baseline and mean follow-up values divided by their mean standard deviations’ (sd) difference. We considered SMR values of less than 0.2 as small, from 0.2 to 0.5 as moderate, and values above 0.8 as large, following Cohen’s criteria [21]. EFA was conducted to explore the overlap in the underlying constructs of the two instruments. If factors have meaningful loadings from the two different instruments (EQ-5D and HIT-6), these instruments are assumed to capture the same underlying latent structure. We considered factor loadings above 0.3 as ‘meaningful’ [22]. For ordered data, the preferred method to determine the number of factors is to conduct parallel analysis with polychoric correlations instead of Pearson correlations [23]. It is believed that Pearson correlations underestimate the relationship between ordered categorical data because of the categorisation [24]. Furthermore, Glorfeld (1995) showed that parallel analysis performs well with non-normally distributed data [25]. The chosen factoring mode was weighted least squares, which makes no distributional assumption, thus being appropriate for ordinal data [26]. Varimax and promax rotations were used to interpret factor loadings.

#### Mapping model development

Since there is no specific model recommended by guidelines on best practices for mapping, we applied several models [12]. As our data contains repeated measurements per individual over time, we accounted for dependencies between observations by including random effects in our models, and estimated mixed-effects linear regression models (fit by maximum likelihood), mixed-effects tobit censored at the upper bound at 1, adjusted limited dependent variable mixture models, mixture beta regression models, and two-part models. For mixed-effects linear models and mixed-effects tobit, we compared models where the overall HIT-6 score versus the HIT-6 several dimensions were used as independent variables. Interaction terms and quadratic terms were considered. Models with the lowest BIC (Bayesian information criterion) were chosen. With regard to the two-part model, in a first stage, a mixed-effects logistic regression is fit to predict the probability of a respondent having full health. In a second stage a mixed-effects linear regression only based on those without full health was estimated. The overall expected EQ-5D index score was calculated using an expected value approach [27].

$$\begin{gathered} E({\text{EQ}} - 5D) = \Pr ({\text{Full}}\;{\text{Health}}) * ({\text{EQ}} - 5\;{\text{in}}\;{\text{Full}}\;{\text{Health}}) \hfill \\ \quad \quad + (1 - \Pr ({\text{Full}}\;{\text{Health}})) * ({\text{EQ}} - 5\;{\text{Not}}\;{\text{in}}\;{\text{Full}}\;{\text{Health}}). \hfill \\ \end{gathered}$$

We also fitted adjusted limited dependent variable mixture models with one to four components, with the Stata command aldvmm, which was specifically developed to deal with health utility data [28]. These models allow to limit the dependent variable to the EQ-5D country-specific range, while taking into account the gap between 1 and the next feasible value (0.974 in the case of Germany). We conducted both adjusted limited dependent variable mixture models and mixture beta regression models with and without the inclusion of this truncation point, as well as with and without the inclusion of a probability mass at full health and at this truncation point (for beta mixture models only). We used the estimated parameters from a constant-only model in our mixture models, to find the global maximum, since mixture models are known to have multiple optima [28]. Unlike in the other models, we could not account for repeated measures by including random effects. We did, however, compute robust cluster-corrected standard errors.

HIT-6 related variables, sex, age, and migraine type were pre-defined as having to be part of the model. Given that mapping algorithms are intended to be used by other researchers, we only considered age and sex^1^ as possible socio-demographic explanatory variables, since they are almost always collected in studies. Studies have shown that age has an impact on the symptoms of people with migraine, e.g. decrease in frequency of photophobia and phonophobia [29]. Migraine also affects three times more women than men, and it is known that fluctuations in female hormones play an important role in this relationship [30, 31]. Since especially in women, the impact of migraines varies with age, we tested whether there is an interaction between age and sex [29]. We also included the information whether patients suffered from episodic or chronic migraines and its interaction with age. Migraine characteristics evolve across time (e.g. the conversion of episodic to chronic migraine), thus the importance of testing the inclusion of an interaction term between migraine type and age [32].

We conducted complete-case analysis based on the following variables: EQ-5D domains, HIT-6 domains, migraine type, age, and sex.

#### Validation

We plotted the observed and predicted EQ-5D values to visualise the models’ performance. Given the lack of external data to conduct external validation, a tenfold cross-validation was carried out to compare the predictions of each model with the actual EQ-5D scores. This method is recommended for small samples [20]. Models’ predictive performance was assessed with root mean squared error (RMSE), mean absolute error (MAE), and *R*^2^, reporting the mean of all 10 cycles.

Statistical analyses were performed using *R* 3.6.3 and Stata 15 [33, 34]. We used additional *R* packages for data handling [35] and plotting [36], repeated measures correlation [37], and factor analysis [38, 39], a Stata package for variable selection [40] (a preliminary version of gsreg 2.0 provided by the authors was used, which allowed its use with a mixed-effects linear regression estimated by maximum-likelihood), the package aldvmm to fit adjusted limited dependent variable mixture models [28], as well as the betamix package for conducting beta mixture regressions [40].

## Results

The dataset used for the analysis contains 1010 observations, based on 410 patients, as 16 patients had missing data, such that 22 out of the 1032 (2.13%) observations had to be removed. thus the dataset used for the analysis contains 1010 observations, based on 410 patients. 7 out of 16 were excluded from the analysis because they did not have full data on other time points.

87.3% of all participants were female, with an average age of 41 years (Table 1).

**Table 1:** Patient characteristics and measurements of EQ-5D and HIT-6 at baseline

	Chronic migraine (132)	Episodic migraine (278)	Total (410)
Age, mean (SD)	40.1 (11.4)	41.5 (12.0)	41.1 (11.8)
Sex (%)^a^			
Female	119 (90.2%)	239 (86.0%)	358 (87.3%)
Male	13 (9.8%)	39 (14.0%)	52 (12.7%)
BMI			
Mean (SD)	25.0 (4.89)	24.6 (4.66)	24.7 (4.74)
Missing	2 (1.5%)	1 (0.4%)	3 (0.7%)
Comorbidities (%)			
Yes	80 (60.6%)	154 (55.4%)	234 (57.1%)
No	49 (37.1%)	121 (43.5%)	170 (41.5%)
Missing	3 (2.3%)	3 (1.1%)	6 (1.5%)
Marital status (%)			
Married	55 (41.7%)	134 (48.2%)	189 (46.1%)
Single	65 (49.2%)	119 (42.8%)	184 (44.9%)
Widowed	1 (0.8%)	4 (1.4%)	5 (1.2%)
Divorced	8 (6.1%)	18 (6.5%)	26 (6.3%)
Missing	3 (2.3%)	3 (1.1%)	6 (1.5%)
Professional qualification (%)			
Other	11 (8.3%)	11 (4.0%)	22 (5.4%)
University^b^	44 (33.3%%)	120 (43.2%)	164 (40.0%)
Without a degree	12 (9.1%)	24 (8.6%)	36 (8.8%)
Apprenticeship	62 (47.0%)	120 (43.2%)	182 (44.4%)
Missing	3 (2.3%)	3 (1.1%)	6 (1.5%)
Officially recognised disability (%)			
Yes	22 (16.7%)	45 (16.2%)	67 (16.3%)
No	107 (81.1%)	231 (83.1%)	338 (82.4%)
Missing	3 (2.3%)	2 (0.7%)	5 (1.2%)
EQ-5D-5L			
Mean utility from − 0.661 to 1 (SD)	0.689 (0.296)	0.842 (0.198)	0.792 (0.244)
VAS mean from 0 to 100 (SD)	58.0 (23.4)	70.7 (20.2)	66.6 (22.1)
HIT-6			
Mean (SD)	65.2 (4.21)	64.4 (4.38)	64.7 (4.33)
Severity level (%)			
Severe impact	2 (1.5%)	10 (3.6%)	12 (2.9%)
Substantial impact	7 (5.3%)	16 (5.8%)	23 (5.6%)
Some impact	122 (92.4%)	251 (90.3%)	373 (91.0%)
Little or no impact	1 (0.8%)	1 (0.4%)	2 (0.5%)

*SD* standard deviation, *VAS* Visual Analog Scale.

^a^In German there are no different terms to define sex versus gender. The term “Geschlecht” can be both understood as sex or gender. In this project, participants filled in their own “Geschlecht”.

^b^Including university of applied sciences

**Fig. 1:**
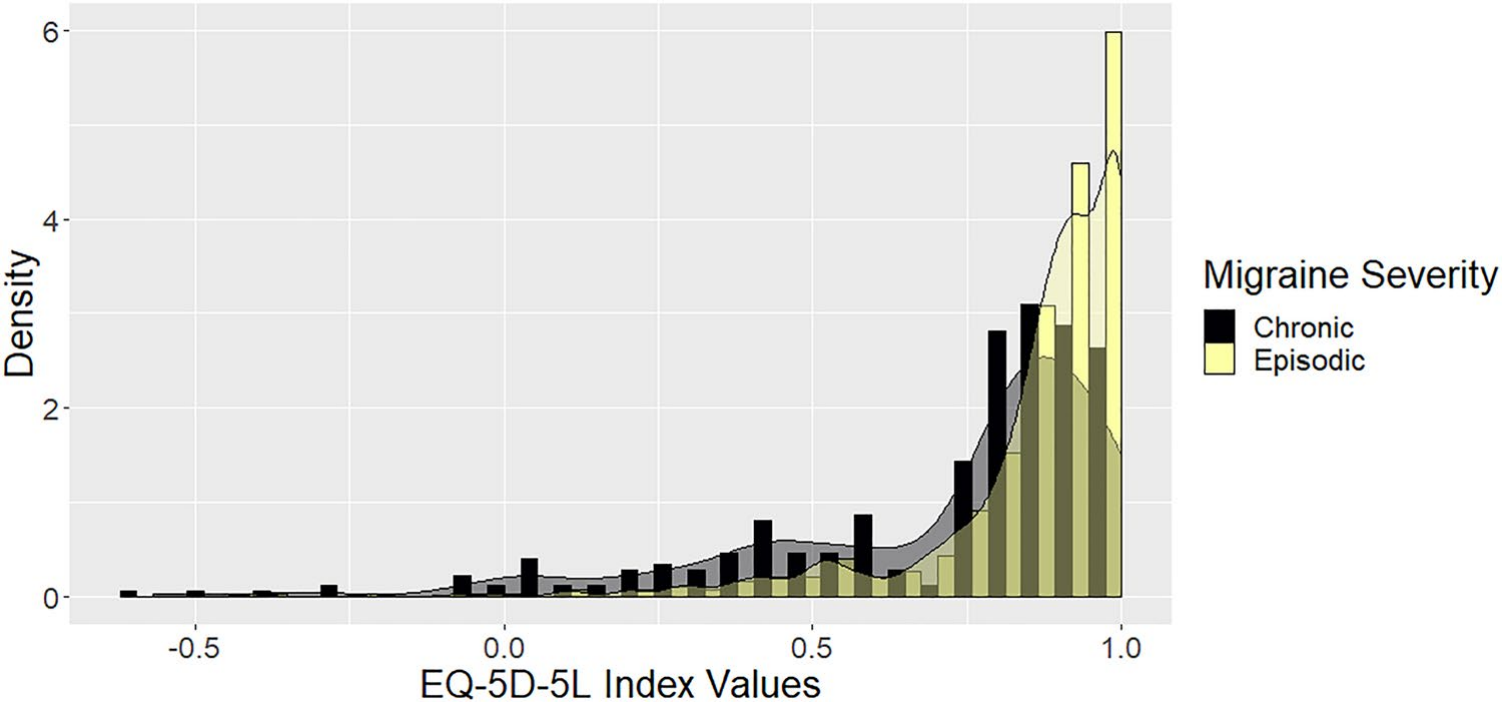
EQ-5D-5L histogram of number of responses histogram and kernel density plot (for episodic vs chronic migraine)

Health utility values derived from EQ-5D ranged from − 0.57 to 1. We observed a ceiling effect in EQ-5D scores, with a skewness of − 2.33 and a kurtosis of 9.45, pointing to a left skew with few negative observations (Fig. 1). Data are considerably more skewed for patients with episodic migraine than for patients with chronic migraine. The mean EQ-5D utility value was 0.82 (sd 0.23) for all patients, 0.86 (sd 0.18) for patients with episodic migraine, and 0.72 (sd 0.30) for patients with chronic migraine.

**Fig. 2:**
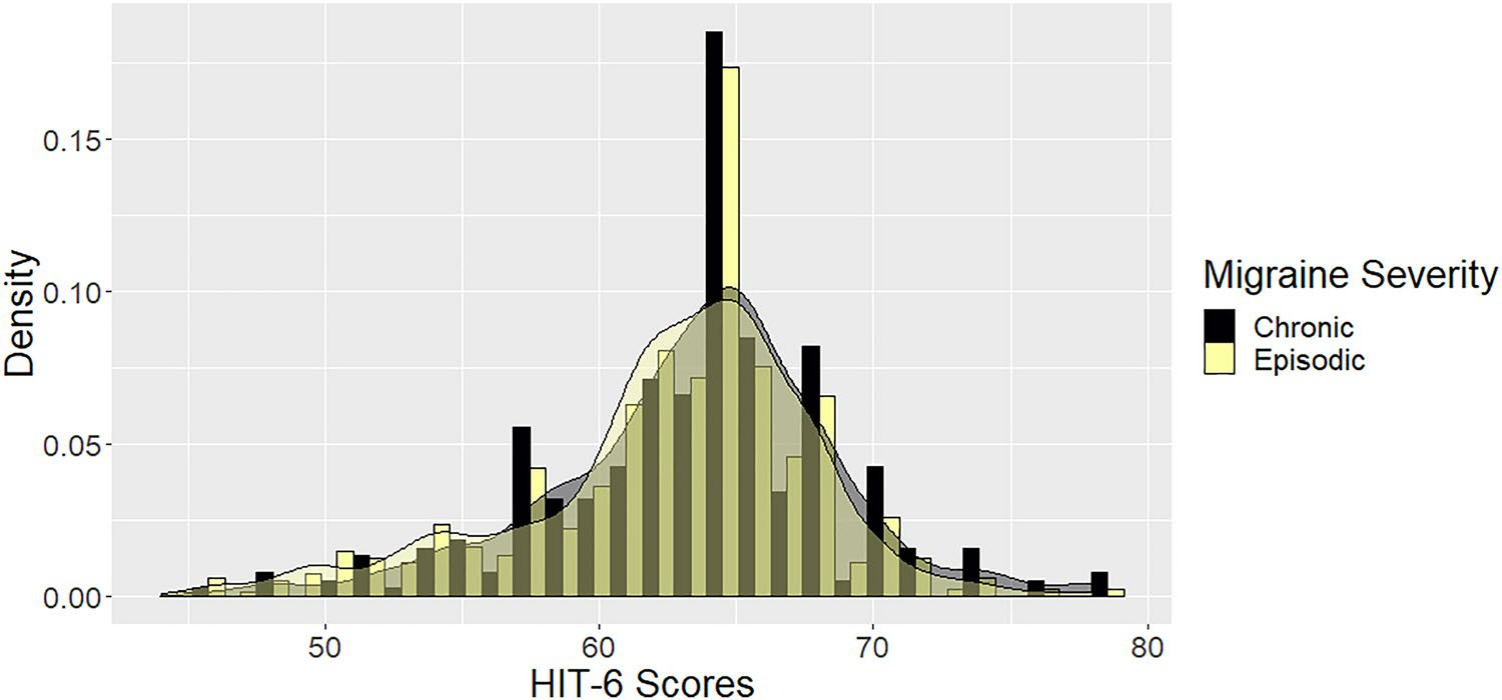
HIT-6 histogram of number of responses histogram and kernel density plot (for episodic vs chronic migraine)

HIT-6 scores ranged from 44 to 78 (possible score range 36–78). The skewness of − 0.64 indicated that the HIT-6 scores are only slightly skewed to the left (Fig. 2). There was no floor effect, no patient had the lowest score possible, and the ceiling effect was small (5 out of 1010 observations; 0.5%).

In EQ-5D, there was no floor effect (proportion of respondents reporting the worst level for all five dimensions), i.e. no patient had the lowest utility score possible (− 0.661 in the German value set). However, the ceiling effect (proportion of participants reporting the best level for all dimensions) amounted to 47.3% (194/410).

### Conceptual overlap

We consider that occupation and daily activities can be measured by the EQ-5D dimension “usual activities” and by questions 2, 3, and 4 from the HIT-6. Physical health is captured by “pain/discomfort” and “mobility” in the EQ-5D, and by question 5 from the HIT-6. Self-care is only measured by the EQ-5D.

The correlation coefficient between EQ-5D score value and the HIT-6 total score amounted to − 0.30. In terms of EQ-5D value and the different HIT-6 dimensions, the coefficients ranged between − 0.153 and − 0.234. The correlation coefficients between each EQ-5D domain and the overall HIT-6 score ranged from 0.077 to 0.300 (Table A.1). Lastly, the correlation coefficients among each domain from the two instruments ranged from 0.021 to 0.227. The highest correlation (0.227) was found between EQ-5D pain/discomfort and HIT-6 q4. See Supplementary Tables A.1, A.2, and A.3 for correlation tables, additionally stratified by migraine severity level.

The EQ-5D total score and the different dimensions show small SRMs, while the HIT-6 total score and its different questions show small to moderate responsiveness. For EQ-5D dimensions, SRM values range from 0.088 to 0.280 and for the HIT-6 from 0.211 to 0.669 (see Supplementary Table A.4). Although the lack of responsiveness may be in part because we are also analysing patients in the control group, this still does not explain why the responsiveness of the HIT-6 is higher than that of the EQ-5D.

We considered three factors in the EFA. Factor 1 had meaningful loadings (i.e. higher than 0.3) on all EQ-5D domains, but not on HIT-6 domains. Factors 2 and 3 loaded only on HIT-6 domains, specifically questions 2–6 for Factor 2, questions 1 and 2 for Factor 3. Considering that this question had a higher loading in Factor 2, thus belonging to this factor, Factor 2 had meaningful loadings in five out of six HIT-6 domains (Table 2). Similarly, using an orthogonal rotation, all EQ-5D items loaded on the same factor, while HIT-6 items loaded on both Factors 2 and 3 (Supplementary Table A.5).

**Table 2:** Summary of the Exploratory Factor Analysis (EFA) results for 3 loadings and their cumulative variance (varimax rotation)

	Factor 1’ loadings	Factor 2’ loadings	Factor 3’ loadings
Mobility	0.749	0.125	0.156
Self-care	0.556		0.110
Daily activities	0.840	0.237	
Pain/discomfort	0.761	0.170	0.115
Anxiety/depression	0.433	0.257	
HIT-6 Q1	0.119	0.225	0.629
HIT-6 Q2	0.190	0.539	0.404
HIT-6 Q3		0.342	0.355
HIT-6 Q4	0.196	0.769	0.239
HIT-6 Q5	0.220	0.684	0.167
HIT-6 Q6	0.208	0.858	0.176
Cumulative variance	0.229	0.449	0.528

Meaningful loadings are underlined (i.e. higher than 0.3)

As the EFA does not correctly take the repeated measurement nature of the data into account, we performed a sensitivity analysis based on baseline data only. The results did not relevantly differ in terms of number of factors and meaningful loadings.

The lack of overlap in all three factors, using the two different types of rotations, suggests that the EQ-5D and the HIT-6 potentially do not capture the same latent constructs.

### Mapping models

Table 3 and the Excel file in the Electronic Supplementary Material present information on the models’ coefficients and their predictive ability. Overall, for the same statistical method, models which included the HIT-6 total score performed better than those which included all HIT-6 questions as independent variables. The inclusion of interaction terms (between age and sex, migraine type and age, and migraine type and age) did not relevantly improve the prediction of EQ-5D scores within any of the six models. On the contrary, the addition of quadratic terms both for HIT-6 overall score and for several HIT-6 dimensions proved to enhance some of the models with regard to their goodness-of-fit. In the two-part model, the first model only included the total HIT-6 score, the type of migraine, age, and sex, the second included the same variables plus the quadratic term of the HIT-6 score.

**Table 3:** Performance measurements and validation results of 10 evaluated mapping models

Model	Specification	Predicted mean	Predicted minimum	Predicted maximum	Cross-validation		
					RMSE	MAE	Pseudo *R*^2^
Actual EQ-5D-5L value	n.a.	0.817	− 0.57	1	n.a.	n.a.	n.a.
Model A	ME Linear	0.8173	0.2488	0.9740	0.1970	0.1380	0.2778
Model B	ME Linear	0.8152	0.3748	1.0704	0.2002	0.1411	0.2558
Model C	ME Tobit	0.8156	0.2376	0.9305	0.1991	0.1366	0.2754
Model D	ME Tobit	0.8004	0.3837	0.9809	0.2046	0.1431	0.2394
Model E	TPM	0.7999	0.1813	0.9469	0.1212	0.1355	0.2843
Model F	TPM	0.7873	0.4389	1.1453	0.1244	0.1424	0.2338
Model G	ALDVMM	0.7882	0.3946	0.9589	0.1992	0.1345	0.2882
Model H	ALDVMM	0.8278	0.4947	0.9797	0.2023	0.1368	0.2593
Model I	BETAMIX with PM at full health	0.8273	0.3910	0.9551	0.1991	0.1347	0.2939
Model J	BETAMIX with PM at full health	0.8259	0.4839	0.9782	0.2018	0.1362	0.2705

*ALDVMM* adjusted limited dependent variable mixture, *BETAMIX* Beta Mixture Model (with inflation), *MAE* mean absolute error, *ME* mixed-effects, *n.a.* not applicable, *PM* probability mass, *RMSE* root mean square error, *SD* standard deviation, *TPM* two-part model

**Fig. 3:**
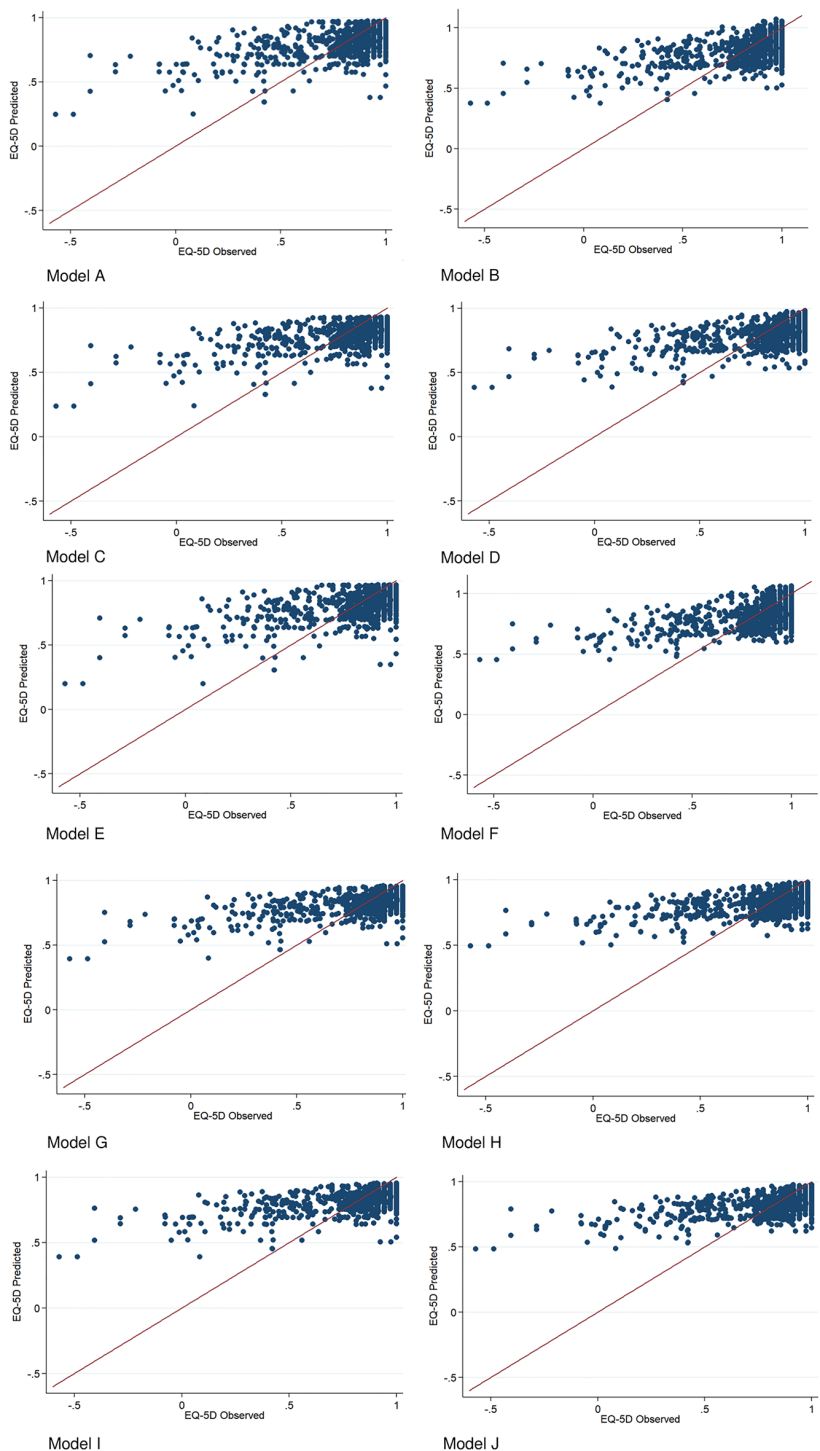
Scatter plots comparing observed vs predicted EQ-5D-5L utility values. Legend—Model **A**: Mixed-effects linear regression, total HIT-6 score. Model **B**: Mixed-effects linear regression, individual HIT-6 questions. Model **C**: Mixed-effects Tobit, total HIT-6 score. Model **D**: Mixed-effects Tobit, individual HIT-6 questions. Model **E**: Two-part model, total HIT-6 score. Model **F**: Two-part model, individual HIT-6 questions. Model **G**: Adjusted limited dependent variable mixture, total HIT-6 score. Model **H**: Adjusted limited dependent variable mixture, individual HIT-6 questions. Model **I**: Beta Mixture Model (with inflation), total HIT-6 score. Model **J**: Beta Mixture Model (with inflation), individual HIT-6 questions

Figure 3 shows the observed and the predicted EQ-5D values for the different models. Our models underestimated utilities for those with poorer health states and overestimated them for those with better health states, as is common in mapping studies [41]. Although linear regression models can yield estimates above 1 (given that there is no upper bound), Model A (mixed-effects linear regression with the total HIT-6 score as an independent variable) did not generate estimates out of the bound. For Model A, the maximum predicted value was 0.98 and for Model B (mixed-effects linear regression with the individual HIT-6 questions as independent variables) 1.07.

No model performed best across all goodness-of-fit measures. Model E (two-part model with the total HIT-6 score as the explanatory variable) performed the best in terms of RMSE (Table 3). Although the *R*^*2*^ value is higher for Model G, this model predicts less well both individuals at full health and those with poorer health states than Model E. The *R*^*2*^ value is also higher for Model I than E, but the latter predicts poorer health states better. The adjusted limited dependent variable mixture models and beta-mixture models took into account the gap between full health and the next feasible health state. However, the low number of observations (4) with the state directly after full health (0.974) may explain why these models did not perform better.

Hence, if researchers wish to estimate utilities from the HIT-6 to be employed in cost-utility analyses, Model E should be the preferred model. The corresponding variance–covariance matrix is available in the Electronic Supplementary Material, in Table A.6, to allow probabilistic sensitivity analysis to be carried out and account for uncertainty. However, we would like to remark that this mapping algorithm should only be used as a last resort.

## Discussion

We aimed to assess whether there is a conceptual overlap between the HIT-6 and the EQ-5D and to present a mapping algorithm for the estimation of the EQ-5D score (with German weights) from the HIT-6 questionnaire, a disease-specific survey widely used in clinical trials with migraine patients. Our study points to major differences in the underlying constructs of the HIT-6 and the EQ-5D. The EQ-5D showed a high ceiling effect and small SRMs across time, whereas the HIT-6 did not show a ceiling effect and had a higher responsiveness. This study also provides a mapping algorithm which can be used to map HIT-6 values to EQ-5D utility values.

We expected some overlap between the two instruments since both have been validated in migraine patients. The strength of association between the instruments measured with correlation coefficients was only low to moderate—both for the total scores and for each instrument’s individual questions. Furthermore, the EFA showed that the HIT-6 and EQ-5D do not have a sufficient conceptual overlap and potentially estimate different underlying constructs. There are several reasons that might explain the lack of overlap. First, the recall period in the instruments’ questions is different. While all EQ-5D questions refer to the day the questionnaire is filled out, three questions in the HIT-6 refer to the previous 4 weeks. Second, the HIT-6 has frequency response categories (ranging from never to always), while the EQ-5D has response categories based on levels of severity. Third, the specificities of both the EQ-5D and the HIT-6 may also play a role. A criticism of the use of the EQ-5D to describe health utilities in patients with migraine is the fact that the survey is conducted at random points in time, thus not differentiating whether or not patients were having a migraine attack at the moment they filled out the survey [42]. The 47.3% participants with level 1 for all five dimensions (ceiling effect) may indicate that the EQ-5D poorly discriminates within patients with migraine. To our knowledge, only two studies validated the use of HIT-6 in German patients with chronic migraine. Although the study by Rendas-Baum et al. [43] included German patients, the authors could not carry out country-specific assessments because of an insufficient sample size of the four European countries included. Thus, they treated the data as one group. Another study by Martin et al. [44] evaluated whether the German version of the HIT-6 is comparable to the United States English HIT-6. Unfortunately, there is no information whether the recruited patients suffered from episodic or chronic migraines. Thus, further research on the validation of HIT-6 in German patients who suffer from episodic and chronic migraine could help explain the lack of conceptual overlap between this questionnaire and the EQ-5D.

Given the lack of responsiveness, as well as the substantial ceiling effect of the EQ-5D for migraine patients, economic evaluations with these patients should consider other approaches to determine value, not necessarily QALYs obtained from generic utility-based instruments. In fact, the guidelines of the International Headache Society state that QALYs may fail to account for specific patient preferences due to the insensitive nature of utility instruments [8]. Thus, the use of QALYs may not be appropriate, even where utility values were collected in the study and no mapping algorithm has to be used. Using clinical effectiveness endpoints (such as monthly migraine days) to conduct cost-effectiveness analyses may thus be more suitable for economic evaluations for migraine. However, these analyses with disease-specific outcomes would pose a different problem, as they do not allow decision-makers to compare resource allocation across different conditions.

Strengths of our study include the fact that trained migraine neurologists provided the migraine diagnosis to the study participants’ and the low percentage of missing data. Furthermore, we could use multiple observations per person and evaluated this data with methods suitable for repeated measurements where possible. The conceptual overlap of EQ-5D and HIT-6 was evaluated carefully prior to investigating mapping algorithms, where the latter were carried out with a broad set of multivariable modelling approaches.

A limitation of our study is that no external validation could be carried out since no dataset containing both EQ-5D answers and HIT-6 was available. Randomised controlled trials are often considered the ‘gold standard’ for evidence-based medicine [45], and although they have several strengths in comparison to other designs, their estimates may lack generalisability with respect to different settings [46]. The ISPOR Task Force Report on Mapping mentions that such trials frequently include less diverse patients than observational studies, due to their inclusion criteria, as well as their limited follow-up [12]. Thus, we have compared some socio-demographic characteristics of our study population to those of migraine patients from a study from the German Migraine and Headache Society, which included 7417 adults from three regions in Germany (see Supplementary Table A.7) [47]. The mean ages reported for episodic migraine were 47.5 (Dortmund Health Study), 50.0 (KORA Augsburg Study), and 50.1 (SHIP Study). For episodic migraine (excluding medication overuse headache, an exclusion criterion of our study) age values were 60.8 in the KORA Augsburg Study and 61.0 in the SHIP Study (no values were available for the Dortmund Health Study). In our study, the mean age was somewhat younger at 40.1 for chronic migraine and 41.5 for episodic migraine, which can be explained by the fact that participants need to have some affinity for using apps and because Berlin is the federal state with the second lowest average age [48]. In terms of sex distribution in the episodic migraine population, the Dortmund Health Study reported 78.7% women, the KORA study 84.2%, and the SHIP 85.6%. The proportion of women in our study was comparable with 86% of participants with episodic migraine. It should be also highlighted that many of those suffering from migraine never seek professional care, such that their characteristics may not be reported in the literature. In Germany, only about two thirds of those suffering from migraine consult a physician to receive treatment [49]. Response mapping models were not conducted, since this method requires many observations in each response category and this dataset contained few responses in the worst levels [50]. The EFA was conducted without taking repeated measurements into account. However, in the sensitivity analysis with baseline data only, we obtained the same results, in terms of number of factors and meaningful loadings. As in other mapping studies, compared to observed EQ-5D, mapped EQ-5D values underestimate scores for those with ‘perfect’ health and overestimate scores for those with worse health states [51]. We ran mixed-effects models with random intercepts only (i.e. different intercepts for each cluster), hence assuming that the association between the independent and dependent variables is highly similar across clusters. Unfortunately, it is not possible to introduce random effects in the adjusted limited dependent variable mixture models.

## Conclusion

Our results suggest that the German versions of EQ-5D and the HIT-6 are not measuring the same underlying concepts due to conceptual differences. Therefore, mapping algorithms shall only be used as a last resort for estimating utilities to be employed in cost-utility analyses.

## Supplementary Information

Below is the link to the electronic supplementary material.

Supplementary file1 (XLSX 16 KB)

Supplementary file2 (DOCX 29 KB)

## Data Availability

The datasets generated and/or analysed during the current study are not publicly available due to data protection reasons.

## Footnotes

1 In German, there are no different terms to define sex versus gender. The term “Geschlecht” can be both understood as sex or gender. In this project, participants filled in their own “Geschlecht”.

## Code availability (software application or custom code)

The code used to conduct the current study is available from the corresponding author on reasonable request.

## References

[1] Radtke A, Neuhauser H (2009). Prevalence and burden of headache and migraine in Germany. Headache.

[2] Buse DC, Manack A, Serrano D (2010). Sociodemographic and comorbidity profiles of chronic migraine and episodic migraine sufferers. J. Neurol. Neurosurg. Psychiatry.

[3] Blumenfeld AM, Varon SF, Wilcox TK (2011). Disability, HRQoL and resource use among chronic and episodic migraineurs: results from the International Burden of Migraine Study (IBMS). Cephalalgia.

[4] Bloudek LM, Stokes M, Buse DC (2012). Cost of healthcare for patients with migraine in five European countries: results from the International Burden of Migraine Study (IBMS). J. Headache Pain.

[5] Stewart WF, Wood GC, Razzaghi H (2008). Work impact of migraine headaches. J. Occup. Environ. Med..

[6] Drummond MF, Sculpher MJ, Claxton K (2015). Methods for the economic evaluation of health care programmes.

[7] EuroQol Research Foundation. EQ-5D-5L User Guide, 2019. Available from: https://euroqol.org/publications/user-guides.

[8] Diener HC, Ashina M, Durand-Zaleski I (2021). Health technology assessment for the acute and preventive treatment of migraine: a position statement of the International Headache Society. Cephalalgia.

[9] Diener H-C, Bussone G, Van Oene JC (2007). Topiramate reduces headache days in chronic migraine: a randomized, double-blind, placebo-controlled study. Cephalalgia.

[10] Lipton RB, Rosen NL, Ailani J (2016). OnabotulinumtoxinA improves quality of life and reduces impact of chronic migraine over one year of treatment: pooled results from the PREEMPT randomized clinical trial program. Cephalalgia.

[11] Kosinski M, Bayliss MS, Bjorner JB (2003). A six-item short-form survey for measuring headache impact: the HIT-6. Qual. Life Res..

[12] Wailoo AJ, Hernandez-Alava M, Manca A (2017). Mapping to estimate health-state utility from non-preference-based outcome measures: an ISPOR good practices for outcomes research task force report. Value Health.

[13] Gillard PJ, Devine B, Varon SF (2012). Mapping from disease-specific measures to health-state utility values in individuals with migraine. Value Health.

[14] Gerlinger C, Bamber L, Leverkus F (2019). Comparing the EQ-5D-5L utility index based on value sets of different countries: impact on the interpretation of clinical study results. BMC Res. Notes.

[15] Lamu AN, Chen G, Gamst-Klaussen T, Olsen JA (2018). Do country-specific preference weights matter in the choice of mapping algorithms? The case of mapping the Diabetes-39 onto eight country-specific EQ-5D-5L value sets. Qual. Life Res..

[16] Connelly M, Rapoff MA, Thompson N, Connelly W (2005). Headstrong: a pilot study of a CD-ROM intervention for recurrent pediatric headache. J. Pediatr. Psychol..

[17] Sorbi MJ, Kleiboer AM, van Silfhout HG (2014). Medium-term effectiveness of online behavioral training in migraine self-management: a randomized trial controlled over 10 months. Cephalalgia.

[18] Hedborg K, Muhr C (2011). Multimodal behavioral treatment of migraine: an internet-administered, randomized, controlled trial. Ups. J. Med. Sci..

[19] Devineni T, Blanchard EB (2005). A randomized controlled trial of an internet-based treatment for chronic headache. Behav. Res. Ther..

[20] Kuhn M, Johnson K (2013). Over-fitting and model tuning. Applied predictive modeling.

[21] Cohen J (2013). Statistical Power Analysis for the Behavioral Sciences.

[22] Tabachnick BG, Fidell LS (2001). Using multivariate statistics.

[23] Holgado-Tello F, Moscoso S, Barbero-García I, Vila E (2010). Polychoric versus pearson correlations in exploratory and confirmatory factor analysis with ordinal variables. Qual. Quant..

[24] Yang Y, Xia Y (2015). On the number of factors to retain in exploratory factor analysis for ordered categorical data. Behav. Res. Methods.

[25] Glorfeld LW (1995). An improvement on horn’s parallel analysis methodology for selecting the correct number of factors to retain. Educ. Psychol. Measure..

[26] Li C-H (2016). Confirmatory factor analysis with ordinal data: Comparing robust maximum likelihood and diagonally weighted least squares. Behav. Res. Methods.

[27] Young TA, Mukuria C, Rowen D (2015). Mapping functions in health-related quality of life: mapping from two cancer-specific health-related quality-of-life instruments to EQ-5D-3L. Med. Decis. Making.

[28] Alava MH, Wailoo A (2015). Fitting adjusted limited dependent variable mixture models to EQ-5D. Stat. J..

[29] Kelman L (2006). Migraine changes with age: IMPACT on migraine classification. Headache.

[30] Peterlin BL, Gupta S, Ward TN, Macgregor A (2011). Sex matters: evaluating sex and gender in migraine and headache research. Headache.

[31] Pistoia F, Sacco S, Maassen van den Brink A, MacGregor EA (2019). Migraine and use of combined hormonal contraception. Gender and migraine.

[32] Andreou AP, Edvinsson L (2019). Mechanisms of migraine as a chronic evolutive condition. J. Headache Pain.

[33] R Core Team (2017). R: A language and environment for statistical computing.

[34] StataCorp LLC (2017). Stata statistical software: release 15 (2017).

[35] Wickham, H., Francois, R., Henry, L., & Müller, K.: dplyr: A grammar of data manipulation. R package version 0.4, 3, p156 (2015)

[36] Wickham H (2016). ggplot2: Elegant graphics for data analysis.

[37] Bakdash JZ, Marusich LR (2017). Repeated measures correlation. Front. Psychol..

[38] Fox, J.: polycor: Polychoric and Polyserial Correlations. R package version 0.7–10. https://CRAN.R-project.org/package=polycor (2019)

[39] Revelle, W. R.: psych: Procedures for Personality and Psychological Research. Software (2017)

[40] Gluzmann, P., Panigo, D.: GSREG: Stata module to perform Global Search Regression. https://EconPapers.repec.org/RePEc:boc:bocode:s457737 (2013)

[41] Brazier JE, Yang Y, Tsuchiya A (2010). A review of studies mapping (or cross walking) non-preference based measures of health to generic preference-based measures. Eur. J. Health Econ..

[42] Xu R, Insinga RP, Golden W, Hu XH (2011). EuroQol (EQ-5D) health utility scores for patients with migraine. Qual. Life Res..

[43] Rendas-Baum R, Yang M, Varon SF (2014). Validation of the Headache Impact Test (HIT-6) in patients with chronic migraine. Health Qual. Life Outcomes.

[44] Martin M, Blaisdell B, Kwong JW, Bjorner JB (2004). The Short-Form Headache Impact Test (HIT-6) was psychometrically equivalent in nine languages. J. Clin. Epidemiol..

[45] Jones DS, Podolsky SH (2015). The history and fate of the gold standard. Lancet.

[46] Sculpher MJ, Claxton K, Drummond M, McCabe C (2006). Whither trial-based economic evaluation for health care decision making?. Health Econ..

[47] Straube A, Pfaffenrath V, Ladwig K-H (2010). Prevalence of chronic migraine and medication overuse headache in Germany—the German DMKG headache study. Cephalalgia.

[48] Statista Durchschnittsalter der Bevölkerung in Deutschland nach Bundesländern* im Jahr 2018. https://de.statista.com/statistik/daten/studie/1093993/umfrage/durchschnittsalter-der-bevoelkerung-in-deutschland-nach-bundeslaendern/. Accessed 7 Mar 2020

[49] Schmerzklinik Kiel (2009) Wer leidet? https://schmerzklinik.de/service-fuer-patienten/migraene-wissen/wer-leidet/. Accessed 13 Jul 2020

[50] Gray LA, Hernández Alava M, Wailoo AJ (2018). Development of methods for the mapping of utilities using mixture models: mapping the AQLQ-S to the EQ-5D-5L and the HUI3 in patients with asthma. Value Health.

[51] Oppe M, Devlin N, Black N (2011). Comparison of the underlying constructs of the EQ-5D and Oxford Hip Score: implications for mapping. Value Health.

