# Supplementary material for: HIT-6 and EQ-5D-5L in patients with migraine: assessment of common latent constructs and development of a mapping algorithm
Source: Eur J Health Econ. 2021 Jul 10;23(1):47–57. doi: 10.1007/s10198-021-01342-9 (PMC8882092; doi:10.1007/s10198-021-01342-9)
Supplement: Supplementary file 2 — Supplementary file2 (DOCX 29 KB) [file 10198_2021_1342_MOESM2_ESM.docx]

**Appendix**

Table A.1 Repeated measurements correlation matrix between EQ-5D-5L, HIT-6 and their domains

| HIT-6/EQ-5D-5L | Mobility | Self-care | Usual activity | Pain/  Discomfort | Depression/ Anxiety | EQ-5D-5L index |
| --- | --- | --- | --- | --- | --- | --- |
| HIT-6 Q1 | 0.095 | 0.067 | 0.130 | 0.115 | 0.080 | -0.153 |
| HIT-6 Q2 | 0.107 | 0.086 | 0.150 | 0.202 | 0.111 | -0.188 |
| HIT-6 Q3 | 0.118 | 0.021 | 0.151 | 0.183 | 0.059 | -0.161 |
| HIT-6 Q4 | 0.144 | 0.023 | 0.193 | 0.227 | 0.112 | -0.234 |
| HIT-6 Q5 | 0.142 | 0.063 | 0.194 | 0.219 | 0.147 | -0.220 |
| HIT-6 Q6 | 0.122 | 0.060 | 0.211 | 0.225 | 0.120 | -0.223 |
| HIT-6 total score | 0.184 | 0.077 | 0.262 | 0.300 | 0.160 | -0.30 |

Table A.2 Correlation table (adjusted for repeated measurements) for chronic migraine patients

| HIT-6/ EQ-5D-5L | Mobility | Self-care | Usual activity | Pain/  Discomfort | Depression/ Anxiety | EQ-5D-5L index |
| --- | --- | --- | --- | --- | --- | --- |
| HIT-6 Q1 | 0.137 | 0.087 | 0.179 | 0.122 | 0.056 | -0.184 |
| HIT-6 Q2 | 0.129 | 0.154 | 0.210 | 0.294 | 0.185 | -0.316 |
| HIT-6 Q3 | 0.188 | 0.034 | 0.158 | 0.214 | 0.145 | -0.208 |
| HIT-6 Q4 | 0.170 | 0.058 | 0.256 | 0.317 | 0.125 | -0.335 |
| HIT-6 Q5 | 0.099 | 0.095 | 0.302 | 0.287 | 0.135 | -0.286 |
| HIT-6 Q6 | 0.129 | 0.119 | 0.312 | 0.368 | 0.087 | -0.349 |
| HIT-6 total score | 0.211 | 0.134 | 0.366 | 0.412 | 0.182 | -0.428 |

Table A.3 Correlation table (adjusted for repeated measurements) for episodic migraine patients

| HIT-6/ EQ-5D-5L | Mobility | Self-care | Usual activity | Pain/  Discomfort | Depression/ Anxiety | EQ-5D-5L index |
| --- | --- | --- | --- | --- | --- | --- |
| HIT-6 Q1 | 0.071 | 0.054 | 0.105 | 0.112 | 0.094 | -0.135 |
| HIT-6 Q2 | 0.096 | 0.041 | 0.122 | 0.155 | 0.073 | -0.114 |
| HIT-6 Q3 | 0.083 | 0.013 | 0.148 | 0.169 | 0.018 | -0.137 |
| HIT-6 Q4 | 0.128 | -0.006 | 0.161 | 0.175 | 0.105 | -0.167 |
| HIT-6 Q5 | 0.168 | 0.042 | 0.143 | 0.183 | 0.153 | -0.184 |
| HIT-6 Q6 | 0.120 | 0.022 | 0.166 | 0.156 | 0.136 | -0.156 |
| HIT-6 total score | 0.1715242 | 0.039 | 0.213 | 0.239 | 0.150 | -0.226 |

Table A.4 EQ-5D-5L and HIT-6 responsiveness measured with Standardised Response Means

|  | Baseline – 3 months | Baseline – 6 months | Baseline – 9 months | Baseline – 12 months |
| --- | --- | --- | --- | --- |
| EQ-5D-5L | 0.221 | 0.144 | 0.194 | 0.104 |
| Mobility | 0.148 | 0.119 | 0.189 | 0.161 |
| Self-care | 0.112 | 0.122 | 0.107 | 0.088 |
| Daily activities | 0.231 | 0.160 | 0.260 | 0.161 |
| Pain/discomfort | 0.188 | 0.161 | 0.170 | 0.133 |
| Anxiety/ depression | 0.229 | 0.192 | 0.280 | 0.230 |
| HIT-6 | 0.540 | 0.585 | 0.621 | 0.706 |
| HIT-6 Q1 | 0.392 | 0.339 | 0.474 | 0.544 |
| HIT-6 Q2 | 0.298 | 0.351 | 0.354 | 0.451 |
| HIT-6 Q3 | 0.211 | 0.282 | 0.298 | 0.360 |
| HIT-6 Q4 | 0.439 | 0.497 | 0.526 | 0.612 |
| HIT-6 Q5 | 0.381 | 0.445 | 0.426 | 0.494 |
| HIT-6 Q6 | 0.553 | 0.520 | 0.662 | 0.669 |

Legend - Standardised Response Means are calculated as the ratio of the difference in the mean baseline and mean follow-up values divided by their mean standard deviations’ difference

Table A.5 Summary of the Exploratory Factor Analysis (EFA) results for 3 loadings and their cumulative variance (promax rotation)

|  | Factor 1’ Loadings | Factor 2’ Loadings | Factor 3’ Loadings |
| --- | --- | --- | --- |
| Mobility | 0.787 |  | 0.107 |
| Self-care | 0.598 | -0.115 |  |
| Daily activities | 0.848 |  |  |
| Pain/discomfort | 0.784 |  |  |
| Anxiety/ depression | 0.391 | 0.242 | -0.172 |
| HIT-6 Q1 |  |  | 0.657 |
| HIT-6 Q2 |  | 0.462 | 0.306 |
| HIT-6 Q3 |  | 0.275 | 0.313 |
| HIT-6 Q4 |  | 0.810 |  |
| HIT-6 Q5 |  | 0.728 |  |
| HIT-6 Q6 |  | 0.942 |  |
| Cumulative Variance | 0.225 | 0.448 | 0.510 |

Meaningful loadings are underlined

Table A.6 Variance-covariance matrix for Model E (two-part model, total HIT-6 score)

|  | HIT-6 score | HIT-6 score^2^ | Age | Male | Episodic migraine | Constant |
| --- | --- | --- | --- | --- | --- | --- |
| HIT-6 score | 0.00042787 |  |  |  |  |  |
| HIT-6 score^2^ | -3.447e-06 | 2.790e-08 |  |  |  |  |
| Age | 5.971e-08 | -1.182e-09 | 5.757e-07 |  |  |  |
| Male | -0.00001208 | 1.334e-07 | -1.662e-06 | 0.00079692 |  |  |
| Episodic migraine | -0.0000121 | 1.121e-07 | -1.029e-06 | -0.00004495 | 0.00036985 |  |
| Constant | 9.272e-06 | -7.890e-08 | 8.066e-08 | -1.379e-06 | -1.431e-06 | -0.00027076 |

Table A.7 Characteristics of participants of several migraine studies in Germany, separately by migraine type

|  | Episodic migraine | | | | Chronic migraine | | | |
| --- | --- | --- | --- | --- | --- | --- | --- | --- |
|  | SMARTGEM | Dortmund | KORA | SHIP | SMARTGEM | Dortmund | KORA | SHIP |
| Mean age | 41.5 | 47.5 | 50.0 | 50.1 | 40.1 | n.a. | 60.8 | 61.0 |
| Women, % | 86.0 | 78.7 | 84.2 | 95 | 90.2 | n.a. | 100 | 100 |
| Mean BMI | 24.6 | 26.8 | 26.6 | 27.3 | 25.0 | n.a. | 28.6 | 31.1 |
| History of diabetes, % | 0.7 | 6.7 | 3.9 | 3.6 | 1.5 | n.a. | 0 | 0 |
| History of hypertension, % | 7.6 | 25.8 | 36.0 | 45.1 | 6.8 | n.a. | 75 | 100 |
| History of myocardial infarction, % | 1.8* | 1.1 | 1.0 | 2.7 | 3.8 * | n.a. | 0 | 50 |

No values were available for the Dortmund Health Study, only for a group of chronic migraine sufferers, which also included patients with medication overuse headache.

* Herzerkrankungen (heart diseases)
